# Bringing Vitamin D and the Vitamin D Receptor into the Limelight

**DOI:** 10.3390/biom14091094

**Published:** 2024-08-31

**Authors:** Jun Sun

**Affiliations:** 1 Division of Gastroenterology and Hepatology, Department of Medicine, University of Illinois Chicago, Chicago, IL 60612, USA; junsun7@uic.edu; 2 UIC Cancer Center, University of Illinois Chicago, Chicago, IL 60612, USA; 3 Jesse Brown VA Medical Center, Chicago, IL 60612, USA

Classically, vitamin D is known to regulate skeletal and mineral ion homeostasis. The active form of vitamin D, 1α,25-dihydroxyvitamin D_3_ [1,25(OH)_2_D_3_], binds with high affinity to the vitamin D receptor (VDR) to perform vitamin D’s biological functions. However, it is now becoming clear that vitamin D and the VDR have a much broader role in human health and diseases [1,2,3]. On 3 June 2024, a new Endocrine Society Clinical Practice Guideline for vitamin D for the prevention of disease was published [4]. It recommends, for the first time, empiric vitamin D supplementation in children and adolescents aged 1 to 18 years to prevent nutritional rickets and potentially lower the risk of respiratory tract infections. It also recommends that adults with high-risk prediabetes, in addition to lifestyle modification, use empiric vitamin D supplementation to reduce the risk of progression to diabetes [4]. This guideline reflects the increasing evidence of the vitamin D/VDR in innate immunity, metabolism, infection, and other diseases [2,5,6]. Vitamin D and the VDR are also known to determine microbiome diversity, eliminate pathogenic bacteria, have anti-inflammatory properties, and inhibit cancer development [7,8,9,10,11]. Thus, in the long run, knowing the mechanisms leading to vitamin D/VDR deficiency will pave the way to a better understanding of the progression of various diseases and the development of novel therapeutic strategies against diseases [12,13].

This Special Issue was initiated in 2023, aiming to discuss the recent progress on vitamin D’s biological effects, molecular mechanisms, and potential therapeutic roles (https://www.mdpi.com/journal/biomolecules/special_issues/vitamin_D_analogues (accessed on accessed on 18 August 2020)). It includes seven original research papers and one review from experts in the field, providing the reader with advances in the understanding of how the vitamin/VDR operates via gene mutation, alteration of vitamin D signaling pathway, VDR protein regulation, and host–microbiome interactions, in healthy or pathophysiological status. These articles also show the recently developed therapeutic strategies in pediatric and adult diseases.

Vitamin D is found naturally in certain foods (e.g., mushrooms and fish). Vitamin D is also available as a dietary supplement. Our body produces vitamin D through skin exposure to sunlight. Thus, vitamin D plays an important role in skin health and disease. The research by Elmelid et al. [14] tested the hypothesis that phototherapy in psoriasis patients would increase both total and free 25(OH)D levels and that free 25(OH)D levels would have a stronger correlation with disease severity than total 25(OH)D. They measured the serum levels of free and total 25(OH)D in psoriasis patients before and after narrow-band UVB (NB-UVB) phototherapy. NB-UVB phototherapy is an effective treatment for mild to severe plaque psoriasis. NB-UVB increased both total and free 25(OH)D levels, suggesting the bioavailability of skin-produced vitamin D. In patients with atopy, Bastyte et al. [15] reported the association between the ***VDR*** gene polymorphisms rs2228570, rs731236, and rs11168293 and vitamin D, total IgE, and blood eosinophils. The regulatory pathways in the pathogenesis of atopic diseases, involving vitamin D, VDR genetic variations, eosinophils, and IgE, underscore the multifaceted nature of vitamin D’s role in immune modulation [16]. Fewer *vdr* mutations were reported in terms of their impact on vitamin D/VDR’s function [3]. However, there are long-standing efforts to identify the genetic evidence of VDR polymorphisms in various human diseases, including inflammatory bowel diseases. It needs to be pointed out that the *vdr* gene is the first human gene identify to shape the diversity of gut microbiome [4].

Vitamin D plays a critical role in infant health and child development. Infants are regularly provided with vitamin D liquid formulations. However, studies linking vitamin D supplementation and the microbiome in pediatric diseases are still limited. Here, Zhao et al. [17] reported that infant vitamin D supplement administration may differentially and independently influence infant gut microbiota metabolites. They investigated whether vitamin D supplementation is associated with fecal glycerol and 1,2-propanediol (1,2-PD) in a 3-month-old infant cohort. Vitamin D supplementation was positively associated with fecal 1,2-PD and inversely associated with glycerol. Fecal 1,2-PD and glycerol concentrations were negatively correlated with each other. There are positive correlations between fecal 1,2-PD, *Bifidobacteriaceae*, *Lactobacillaceae*, *Enterobacteriaceae*, and acetate levels. Sharma et al. [18] demonstrated the beneficial role of maternally administered probiotics in young mice, which maintained higher colonic VDR expression than unexposed mice. The enhanced colonic VDR was able to protect young mice from inflammatory stimulation. These findings indicate a potential therapeutic role for microbiome modulation in preventing diseases through the enhancement of VDR signaling.

In the cardiological disease, Szabo et al. [19] reported 25-Hydroxyvitamin D as an independent marker of left ventricular ejection fraction (LVEF) in heart failure with reduced and mildly reduced ejection fraction. They measured the total 25-hydroxyvitamin D (25(OH)D), serum albumin, and uric acid levels, focusing mainly on vitamin D deficiency. Seventy patients with LVEF < 50% were comprehensively evaluated using ECG, echocardiography, lung ultrasound, blood sampling, and the six-minute walk test. 25(OH)D concentrations (17.6 (15.1–28.2) vs. 22.7 (19.5–33.8), *p* = 0.010) were lower in subjects with severely reduced LVEF. 25(OH)D was independently associated with LVEF as well. Reduced bone mineral density (BMD) was associated with a higher risk of fractures, morbidity, and mortality in kidney transplant patients (KTRs); however, there is no consensus on optimal treatment for the reduced BMD in KTRs. Battaglia et al. [20] assessed the effect of cholecalciferol supplementation on BMD over a follow-up period of 2 years in a cohort of long-term KTRs. Supplementation with cholecalciferol ameliorated Z-score and T-score at LV in long-term KTRs who had never been treated with active or inactive vitamin D sterols, bisphosphonates, and calcimimetics. The findings and prospects of vitamin D in erectile dysfunction are summarized in the review by Crafa et al. [21]. It is also indicated that the low quality and heterogeneity of clinical trials evaluating the effects of vitamin D on erectile function and ED-associated comorbidities do not allow for a univocal conclusion, and there is a need for future studies. The current findings support the need to establish accurate vitamin D supplementation schemes and dietary interventions.

The vitamin D metabolites, 25(OH)_2_D_3_ and 1,25(OH)_2_D_3_, are produced by hydroxylation. However, there are alternative metabolic pathways, e.g., the 4-hydroxylation of 25D_3_. The study by Peluso-Iltis et al. [22] aims to investigate the structure–activity relationships of 4-hydroxy derivatives of 1,25D_3_. 1,4α,25D_3_ and 1,4β,25D_3_ are as potent as 1,25D_3_ in regulating the VDR target genes in rat intestinal epithelial cells and in the mouse kidney. Moreover, 4-hydroxy derivatives promote hypercalcemia in mice at a similar dose as that of the parent compound.

We hope that peers will enjoy reading this Special Issue of *Biomolecules*. We emphasize that the roles of vitamin D and the VDR have sex-difference, tissue specificity, and age-dependence [23]. For example, a recent study mechanistically demonstrates the gender-difference and life span related to vitamin D signaling. In females, the removal of germ cells shortened life span, reduced estrogen, and enhanced insulin-like growth factor 1 signaling. Interestingly, germ cell removal in males improved health with increased vitamin D signaling, using Nothobranchius furzeri, a fish model [24]. In a 1-year lifestyle intervention with the Mediterranean diet in patients with obesity and metabolic syndrome, 25(OH)D status is reported to associate with altered gut microbiota composition, diversity, and functionality [25]. It suggests that modulation of both gut microbiota and 25(OH)D levels potentially influences metabolic pathways [25]. The findings presented in the current Issue and the recent progress in the field will help advance the understanding of the vitamin D/VDR and encourage new research aimed at finding novel mechanisms and evidence for precision nutrition [13] and medicine.
